# The “Pearls” of Multidisciplinary Team: Conquering the Uncommon Rosette Rash

**DOI:** 10.1155/2016/5328603

**Published:** 2016-12-13

**Authors:** Nitin Verma, Charles Pickles, Muhammad Amjad Khan

**Affiliations:** ^1^Department of Paediatrics, Darlington Memorial Hospital, County Durham and Darlington NHS Foundation Trust, Darlington, UK; ^2^Department of Paediatric Dermatology, Darlington Memorial Hospital, County Durham and Darlington NHS Foundation Trust, Darlington, UK

## Abstract

Linear IgA disease of childhood (LAD) also known as chronic bullous disease of childhood is an autoimmune disease with IgA deposition at the basement membrane zone leading to a vesiculobullous rash. It has a clinical appearance which frequently is described as resembling “strings of pearls” or rosette-like. Diagnosis is usually clinical but sometimes biopsy is required. Dapsone is widely considered to be the first line therapy in the treatment of LAD. A 5-year-old girl presented with 4-day history of a widespread painful rash and pyrexia. The rash transformed into painful blisters. A recent contact with chickenpox was present. She remained apyrexial but hemodynamically stable and was treated as chickenpox patient with secondary infection. Due to persistent symptoms after repeated attendance she was reviewed by Dermatology team and diagnosed with linear IgA disease also known as chronic bullous disease of childhood. This was based on the presence of blistering rash with rosette appearance and string of pearl lesions. The clinical features of LAD can be difficult to distinguish from more common skin infections. Benefiting from the experience of other multidisciplinary teams can sometimes be a game changer and can lead to the correct diagnosis and treatment.

## 1. Case Presentation

**Figure 1:**
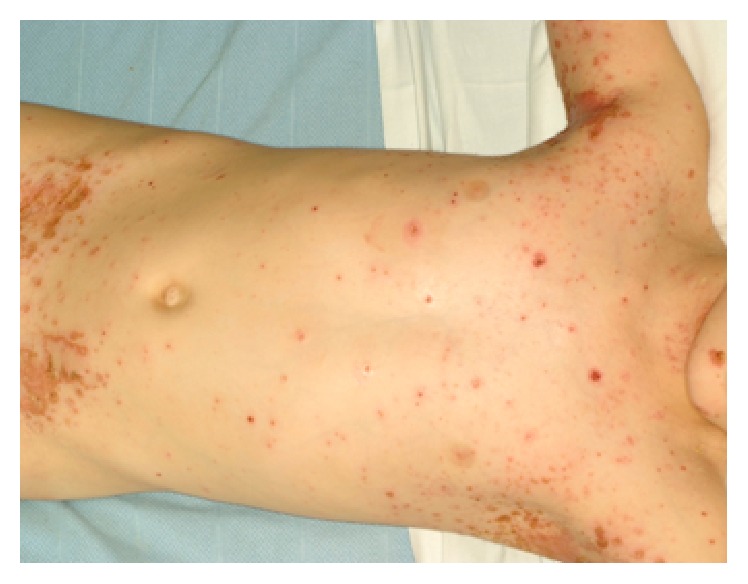
Widespread erythematous papular rash.

A 5-year-old girl presented with 4-day history of a widespread painful rash and pyrexia. The erythematous papular rash started from the retroauricular area and spread all over the body (Figure 1). After 2 days, this rash transformed into painful blisters. A recent contact with chickenpox was also noted.

On admission to paediatric ward, she was apyrexial, in pain, but hemodynamically stable with a widespread erythematous rash and multiple ruptured blisters on her torso and back. She was diagnosed with chickenpox along with a secondary bacterial infection and started on intravenous Clindamycin due to history of penicillin allergy. She was given oral morphine for her pain.

Her blood tests showed CRP 29, WCC 3.9, and a lymphocyte count 1.0. A wound swab and blood culture showed no growth. She received IV antibiotics for 4 days and was then discharged on oral Clindamycin and regular pain relief.

She attended 6 days after the first admission complaining of sore skin under the axilla. As she remained apyrexial and systemically well, she was discharged home with further 5 days of oral Clindamycin. Her wound swab grew a heavy growth of Staphylococcus aureus sensitive to flucloxacillin. Clindamycin was continued.

**Figure 2:**
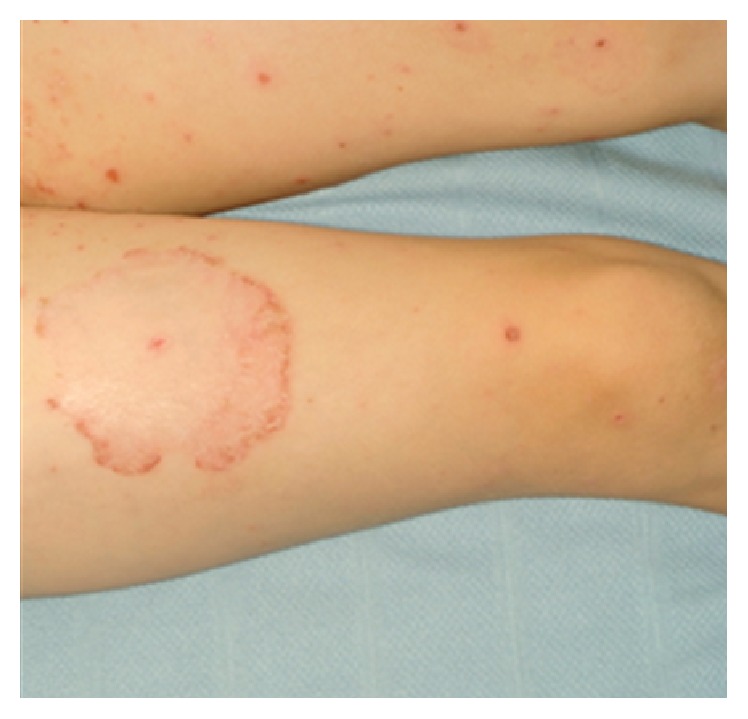
Annular lesion on right thigh with surrounding vesicles with “string of pearls” appearance.

She was readmitted 10 days after her initial presentation with new blistering lesions associated with desquamation in the axilla and groin and over the buttocks. She remained apyrexial and her pain had settled. Intravenous Aciclovir was added after a discussion with Infectious Diseases team, assuming it to be a persistent Varicella Zoster infection. She was reviewed by Dermatology team due to new blistering lesions on Aciclovir and diagnosed with linear IgA disease also known as chronic bullous disease of childhood. This was based on the presence of blistering rash with rosette appearance and string of pearl lesions (Figure 2). She was started on oral Dapsone, potent topical steroid, and a topical antibacterial cream and Aciclovir discontinued. Clindamycin was continued for another week. Her skin lesions quickly got better with no new blistering lesions. On Dapsone, she has had no further blistering lesions. Currently she is on continuing monitoring for Dapsone and will continue to be on it for the foreseeable future. Family were made aware of the side effects of Dapsone. She is having regular blood tests to look out for Dapsone related haemolysis and hepatitis. Three months after initial presentation, pink coloured papules were noted at the sites of blisters on chest and anterior axillary fold and behind the left ear. These were diagnosed as keloid scars. Due to that, she has intralesional steroid injection for these keloid scars at a later date.

## 2. Discussion

Linear IgA disease of Childhood (LAD) also known as chronic bullous disease of childhood is an autoimmune disease with IgA deposition at the basement membrane zone leading to a vesiculobullous rash. It presents with both cutaneous involvement and mucosal involvement. Children classically present with widespread annular blisters that exhibit a predilection for the lower abdomen, thighs, and groin [1]. Face, hands, and feet are rarely involved in children [2]. In our case, there were distinct vesicles on the face which made us consider a diagnosis of chickenpox. New blisters often form at the periphery of resolving lesions, resulting in an annular appearance. Such lesions are frequently described as resembling “strings of pearls” or rosette-like appearance [3]. The vesicles and bullae typically have a tense, rather than flaccid, pemphigus-like quality [4].

Mucosal lesions primarily present as erosions or ulcers; the detection of intact vesicles or bullae is uncommon. Any mucosal surface may be affected though the oral and ocular mucosae are the most commonly affected mucosal sites [5]. Oral lesions usually consist of painful erosions and ulcerations produced by the rupture of blisters or vesicles that are very hard to see due to the continuous trauma and may appear everywhere in the oral cavity [6]. These painful erosions were noted in our case. In a case series of 25 children with LAD in UK, mucosal involvement was present in 64 percent [5]. Affected children may be asymptomatic, but pruritis is common and may be severe. In some patients, intense pruritis indicates recurrences of the disease [2].

Diagnosis of this condition is based on clinical or histopathologic findings. The demonstration of linear deposits of IgA along the basement membrane zone via direct immunofluorescence (DIF) is the gold standard for diagnosis [2]. In our case, the diagnosis was entirely clinical. Rapid clinical benefit to the treatment obviated the need for histopathologic diagnosis.

There are no large, randomized, double-blind, controlled trials on the treatment for LAD. Dapsone is widely considered to be the first line therapy in the treatment of LAD [6, 7]. In a retrospective study in which 19 children received Dapsone as monotherapy, 8 patients (42%) attained lesion regression within a mean duration of 13 days [8]. Although most patients tolerate the drug well, as was with our case, Dapsone must be administered with caution, since potential serious adverse effects, such as haemolysis, methaemoglobinaemia, agranulocytosis, hepatitis, cholestatic jaundice, hypersensitivity syndrome, and peripheral motor neuropathy, may occur as a result of therapy [6]. Haemolysis occurs to some degree in all patients. Regular monitoring of full blood count (FBC) and liver function tests is recommended. Our case is having monthly blood tests. Her blood tests have been within normal limits. She is going to be on Dapsone for the foreseeable future to maintain remission.

Patients who cannot tolerate Dapsone may benefit from treatment with sulphonamide medications that have structural similarities with Dapsone [2]. Patients who fail to respond sufficiently to Dapsone may also derive benefit from the addition of systemic glucocorticoids to Dapsone therapy [9, 10]. However, due to the potential adverse effects of systemic glucocorticoids, these agents are not recommended for long-term use. It has been suggested that topical corticosteroids may be sufficient for disease control in patients with mild or localized LAD [7]. Topical corticosteroids are primarily used as adjunctive therapy [11] as was the situation in our case. LAD typically persists for months to several years prior to spontaneous resolution and resolves in most children prior to puberty. However, disease may persist for a decade or longer, and relapses after long periods of remission may occur [1].

## 3. Conclusion

The clinical features of LAD can be difficult to distinguish from dermatitis herpetiformis and bullous impetigo [4]. In our case, a history of contact with chickenpox and vesicles on the face added weight to a concomitant chickenpox infection. In our case we started off with an appropriate line of treatment with antibiotics to cover super added bacterial infection (confirmed as* Staphylococcus aureus* on swabs) on chickenpox lesions and added in Aciclovir. After minimal clinical benefit, involvement of other multidisciplinary teams (Dermatology and ID) ultimately led us to the correct diagnosis and treatment. It highlights the importance of working closely with other MDT's.

## References

[1] Mintz E. M., Morel K. D. (2011). Clinical features, diagnosis, and pathogenesis of chronic bullous disease of childhood. *Dermatologic Clinics*.

[2] Guide S. V., Marinkovich M. P. (2001). Linear IgA bullous dermatosis. *Clinics in Dermatology*.

[3] Lara-Corrales I., Pope E. (2010). Autoimmune blistering diseases in children. *Seminars in Cutaneous Medicine and Surgery*.

[4] Sansaricq F., Stein S. L., Petronic-Rosic V. (2012). Autoimmune bullous diseases in childhood. *Clinics in Dermatology*.

[5] Wojnarowska F., Marsden R. A., Bhogal B., Black M. M. (1988). Chronic bullous disease of childhood, childhood cicatricial pemphigoid, and linear IgA disease of adults. A comparative study demonstrating clinical and immunopathologic overlap. *Journal of the American Academy of Dermatology*.

[6] Fortuna G., Marinkovich M. P. (2012). Linear immunoglobulin A bullous dermatosis. *Clinics in Dermatology*.

[7] Ng S. Y., Venning V. V. (2011). Management of linear IgA disease. *Dermatologic Clinics*.

[8] Monia K., Aida K., Amel K., Ines Z., Becima F., Ridha K. (2011). Linear IGA bullous dermatosis in Tunisian children: 31 Cases. *Indian Journal of Dermatology*.

[9] Nanda A., Dvorak R., Al-Sabah H., Alsaleh Q. A. (2006). Linear IgA bullous disease of childhood: an experience from Kuwait. *Pediatric Dermatology*.

[10] Kharfi M., Khaled A., Karaa A., Zaraa I., Fazaa B., Kamoun M. R. (2010). Linear IgA bullous dermatosis: the more frequent bullous dermatosis of children. *Dermatology Online Journal*.

[11] Culton D. A., Diaz L. A. (2012). Treatment of subepidermal immunobullous diseases. *Clinics in Dermatology*.

